# Stress-Specific Spatiotemporal Responses of RNA-Binding Proteins in Human Stem Cell-Derived Motor Neurons

**DOI:** 10.3390/ijms21218346

**Published:** 2020-11-06

**Authors:** Jasmine Harley, Rickie Patani

**Affiliations:** 1 Department of Neuromuscular Diseases, Queen Square Institute of Neurology, University College London, London WC1N 3BG, UK; jasmine.harley@crick.ac.uk; 2 Human Stem Cells and Neurodegeneration Laboratory, The Francis Crick Institute, 1 Midland Road, London NW1 1AT, UK

**Keywords:** amyotrophic lateral sclerosis (ALS), motor neurons (MNs), RNA-binding proteins (RBPs), osmotic stress, heat stress, oxidative stress, nuclear, cytoplasmic

## Abstract

RNA-binding proteins (RBPs) have been shown to play a key role in the pathogenesis of a variety of neurodegenerative disorders. Amyotrophic lateral sclerosis (ALS) is an exemplar neurodegenerative disease characterised by rapid progression and relatively selective motor neuron loss. Nuclear-to-cytoplasmic mislocalisation and accumulation of RBPs have been identified as a pathological hallmark of the disease, yet the spatiotemporal responses of RBPs to different extrinsic stressors in human neurons remain incompletely understood. Here, we used healthy induced pluripotent stem cell (iPSC)-derived motor neurons to model how different types of cellular stress affect the nucleocytoplasmic localisation of key ALS-linked RBPs. We found that osmotic stress robustly induced nuclear loss of TDP-43, SPFQ, FUS, hnRNPA1 and hnRNPK, with characteristic changes in nucleocytoplasmic localisation in an RBP-dependent manner. Interestingly, we found that RBPs displayed stress-dependent characteristics, with unique responses to both heat and oxidative stress. Alongside nucleocytoplasmic protein distribution changes, we identified the formation of stress- and RBP-specific nuclear and cytoplasmic foci. Furthermore, the kinetics of nuclear relocalisation upon recovery from extrinsic stressors was also found to be both stress- and RBP-specific. Importantly, these experiments specifically highlight TDP-43 and FUS, two of the most recognised RBPs in ALS pathogenesis, as exhibiting delayed nuclear relocalisation following stress in healthy human motor neurons as compared to SFPQ, hnRNPA1 and hnRNPK. Notably, ALS-causing valosin containing protein (VCP) mutations did not disrupt the relocalisation dynamics of TDP-43 or FUS in human motor neurons following stress. An increased duration of TDP-43 and FUS within the cytoplasm after stress may render the environment more aggregation-prone, which may be poorly tolerated in the context of ALS and related neurodegenerative disorders. In summary, our study addresses stress-specific spatiotemporal responses of neurodegeneration-related RBPs in human motor neurons. The insights into the nucleocytoplasmic dynamics of RBPs provided here may be informative for future studies examining both disease mechanisms and therapeutic strategy.

## 1. Introduction

Amyotrophic lateral sclerosis (ALS) is a fatal neurodegenerative disease characterised by the progressive degeneration of motor neurons (MNs). Disordered RNA processing, regulated by RNA-binding proteins (RBPs), plays a central role in ALS pathogenesis. RBPs are involved in regulating transcription, RNA processing, localisation, function and decay. Many ALS-causing gene mutations encode RBPs, including transactive response DNA-binding protein 43 (TDP-43), fused in sarcoma/translocated in liposarcoma (FUS/TLS or FUS) and heterogeneous nuclear ribonucleoprotein A1 (hnRNPA1) [1,2,3]. Subcellular mislocalisation of RBPs is a pathological hallmark of ALS. Indeed TDP-43 is mislocalised from the nucleus to the cytoplasm in the vast majority of cases [4]. More recently, we reported widespread SFPQ and FUS mislocalisation in various ALS models [5,6]. Furthermore, additional RBPs have been shown to be mislocalised in models of ALS, including heterogeneous nuclear ribonucleoprotein K (hnRNPK), one of the most abundant hnRNPs [7]. Accumulation of RBPs in the cytoplasm likely contributes to the formation of RBP oligomers and fibrillar pathological cytoplasmic inclusions seen in ALS [8,9]. As one RBP alone can bind to thousands of RNA targets, any disturbance has the potential to have a broad and diverse impact on RNA metabolism [10].

RBPs are highly dynamic and enable cells to respond to rapid changes in the environment. In response to cellular stress, RBPs may regulate translational repression or activation by shuttling between cellular subcompartments. Previous studies have determined that TDP-43, FUS, SFPQ, hnRNPA1 and hnRNPK undergo changes in cellular localisation in response to various stressors, but this type of investigation has not been conducted in the cell types most relevant to neurodegenerative disease, and precise molecular mechanisms remain incompletely resolved [11,12,13,14,15,16]. Of the few studies that have been performed specifically addressing this issue, stress-induced translocation has been shown to occur in an RBP-specific manner, as oxidative and heat stressors result in the nuclear export of TDP-43, but not FUS [17]. Furthermore, RBPs have also been shown to mechanistically interact to coordinate a cellular stress response; for example, knockdown of hnRNPK has been shown to prevent TDP-43 recruitment to stress granules [18,19].

Considering the role of the aforementioned RBPs (TDP-43, FUS, SFPQ, hnRNPK and hnRNPA1) in ALS and cellular stress, it is clear that a diverse and complex interplay exists. Furthermore, stress in vivo by cerebral ischemia has been shown to induce the aggregation of RBPs including TDP-43, FUS, hnRNPA1 and SFPQ, displaying clear similarity with protein aggregates in neurodegeneration [20]. Therefore, it is important to understand how different stressors affect the localisation—and ultimately the role—of disease-associated RBPs in otherwise healthy motor neurons. In a developing mouse nervous system, it was noted that two-thirds of RBPs are expressed in a cell-type-specific manner [21], therefore highlighting the importance of using a cell-type-specific model to study disease. Indeed, little is known about whether such RBP stress responses are recapitulated in human neurons.

Against this background, we sought to address the hypotheses that the nucleocytoplasmic distribution of a given RBP in human MNs varies in a stress-specific manner and that different RBPs exhibit different responses in a given context. To this end, we used a human induced pluripotent stem cell (iPSC)-derived MN model to examine the spatial and temporal responses of ALS-linked RBPs in response to different cellular stressors. Here, we report that the RBPs TDP-43, SPFQ, FUS, hnRNPA1 and hnRNPK each exhibit unique nucleocytoplasmic redistribution in a stress-specific manner. We also observe stress- and RBP-specific nuclear and cytoplasmic foci formed in these experimental contexts. Additionally, the kinetics of nuclear relocalisation upon recovery from extrinsic stressors was also both stress- and RBP-specific. Importantly, we reveal that TDP-43 and FUS, two of the most recognised RBPs in ALS pathogenesis, exhibit delayed nuclear relocalisation as compared to SFPQ, hnRNPA1 and hnRNPK. An increased duration of TDP-43 and FUS within the cytoplasm after stress may render the environment more aggregation-prone, which may be poorly tolerated in the context of ALS and related neurodegenerative diseases. When examining the post-stress nuclear relocalisation dynamics of TDP-43 and FUS against an ALS genetic background, we found that VCP mutant ALS MNs recover at the same rate as their control counterparts, suggesting that defects in nuclear import of RBPs are not associated with the early stage of disease recapitulated in iPSC model systems. Cumulatively, our study suggests highly regulated responses by individual RBPs to different stressors and may shed light on how their dynamics in these circumstances might render them vulnerable to cytoplasmic mislocalisation and pathological aggregation in ALS.

## 2. Results

### 2.1. RBPs Reveal Distinct Nucleocytoplasmic Distributions in Human MNs 

**Figure 1:**
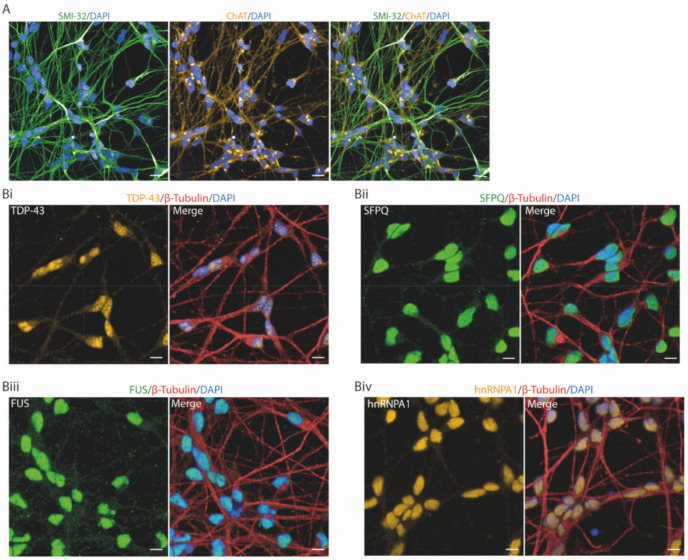

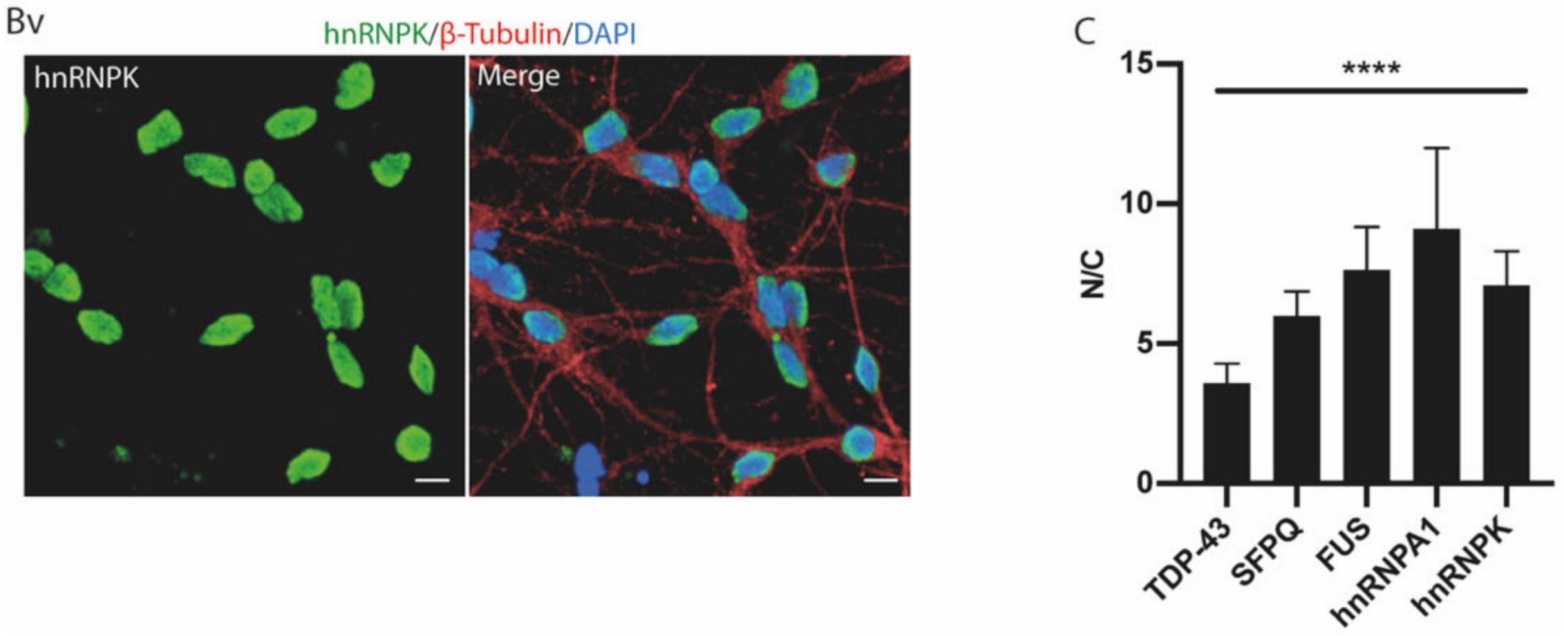
Localisation of amyotrophic lateral sclerosis (ALS)-linked RNA-binding proteins (RBPs) in human induced pluripotent stem cell (iPSC)-derived motor neurons. (**A**) Representative images of human iPSC-derived motor neurons immunolabeled with motor neuron markers SMI-32 and ChAT. Scale bars = 20 μm. (**B**) Representative images of ALS-linked RBPs and neuronal marker β-III-tubulin in iPSC-derived motor neurons. Scale bars = 10 μm. (**Bi**) TDP-43; (**Bii**) SFPQ; (**Biii**) FUS; (**Biv**) hnRNPA1; (**Bv**) hnRNPK. (**C**) The ratio of the average intensity of each RBP immunolabeling in the nucleus and cytoplasm was calculated by automated cell analysis. Data shown are mean nuclear/cytoplasmic ratios (±SD) from >10,000 cells from 3 control lines in 3 independent experimental repeats. Data are plotted per well. **** = *p* < 0.0001, from a Brown–Forsythe ANOVA.

We utilised a highly efficient, characterised and functionally validated method of generating human iPSC-derived spinal cord MNs that we have previously reported [22]. These iPSC-derived MNs were positive for choline acetyltransferase (ChAT), SMI-32 and β-III-tubulin (TUJ1) (Figure 1A,B). Immunocytochemistry of the ALS-linked RBPs, TDP-43, SFPQ, FUS, hnRNPA1 and hnRNPK, in MNs demonstrated a predominantly nuclear localisation (Figure 1B). Single-cell analysis of the nuclear-to-cytoplasmic ratio of >10,000 neurons identified a significant difference between RBPs (Brown–Forsythe ANOVA test, *p* < 0.0001) (Figure 1C). Due to the difficulties defining the cytoplasm of individual neurons, the mean cytoplasmic intensity measurement was taken from a perinuclear region, restricted by a cell mask (Figure S1). The perinuclear mean intensity measurements taken are representative of the neuronal soma. We next examined each RBP for further (qualitative) spatial differences within the nuclear and/or cytoplasmic compartments. All five aforementioned RBPs exhibited predominant nuclear localisation. However, TDP-43 showed heterogeneity within the nucleus, with distinct areas of increased intensity, and hnRNPK exhibited increased localisation at the nuclear membrane. SFPQ, FUS and hnRNPA1 were comparatively homogeneous within the nucleus. TDP-43, SFPQ and FUS were also detected within the soma and neuronal processes, with FUS showing a granular pattern of immunolabeling within the neuronal processes (Figure 1B).

### 2.2. Osmotic Stress Causes RBP-Specific Changes in Nucleocytoplasmic Distribution

**Figure 2:**
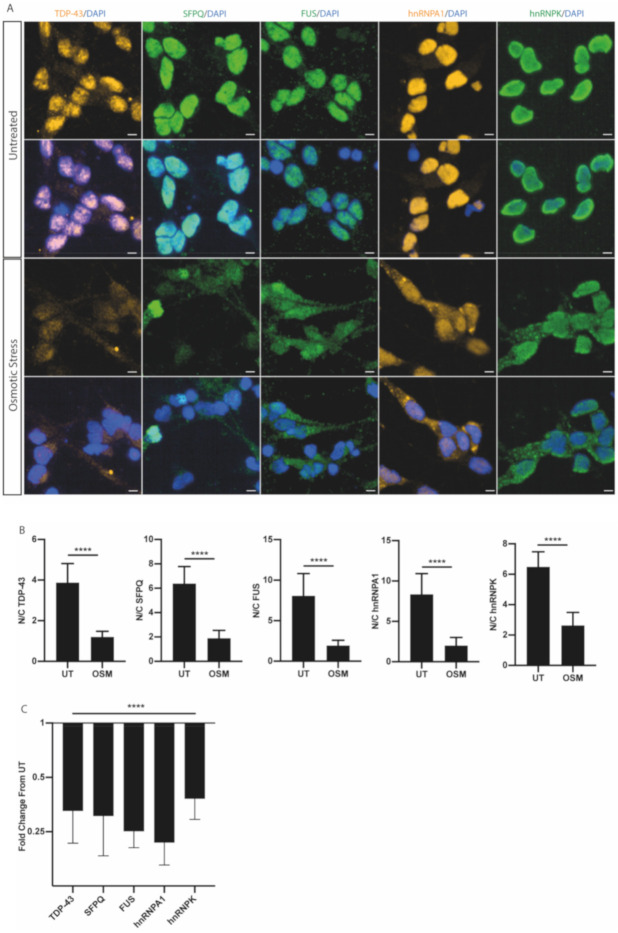
Osmotic stress causes unique changes in the nucleocytoplasmic distributions of ALS-linked RBPs. (**A**) Representative images of TDP-43, SFPQ, FUS, hnRNPA1 and hnRNPK in human motor neurons either untreated or treated with 0.4 M sorbitol treatment for 1 h to induce osmotic stress. Scale bars = 5 μm. (**B**) Quantification of the nuclear/cytoplasmic average intensity ratio for TDP-43, SFPQ, FUS, hnRNPA1 and hnRNPK in untreated (UT) conditions and upon osmotic stress (OSM) show a reduction in the nuclear/cytoplasmic ratio for each RBP upon osmotic stress. Data shown are mean nuclear/cytoplasmic ratios (±SD) per well, calculated from >10,000 cells from 3 control lines in 3 independent experimental repeats as a minimum. *p*-value from unpaired *t*-test; **** = *p* < 0.0001. (**C**) The fold change in the nucleocytoplasmic ratio was calculated upon osmotic stress for each ALS-linked RBP. Data are shown as mean fold change in comparison to untreated values in each experimental repeat. *p*-value is from a Brown–Forsythe ANOVA test; **** = *p* < 0.0001.

We hypothesised that RBPs may exhibit differential responses to osmotic stress. Human motor neurons were treated for 1 h with 0.4 M sorbitol. TDP-43, SFPQ, FUS, hnRNPA1 and hnRNPK all underwent a striking nuclear loss (Figure 2A). Quantification of the nuclear-to-cytoplasmic ratio by individual neuronal analysis demonstrated a significant decrease for each of the five aforementioned RBPs (Figure 2B). However, fold change analyses in the nuclear-to-cytoplasmic ratio indeed demonstrated clear heterogeneity in the effect sizes of localisation responses in an RBP-specific manner (Figure 2C). This is exemplified by hnRNPK exhibiting a relatively modest fold change in the nuclear-to-cytoplasmic ratio (0.38 compared to untreated), compared to FUS and hnRNPA1 (0.25 and 0.22 compared to untreated; Figure 2C). Whole-cell Western blot analysis displayed no difference in total protein levels upon osmotic stress (Figure S2). Furthermore, hyperosmotic stress caused the formation of FUS, hnRNPA1 and hnRNPK cytoplasmic foci (Figure S3A,B). As it has been previously shown that FUS and hnRNPA1 are recruited to stress granules (SGs) upon hyperosmotic stress, we examined colocalisation with an SG marker [11,23]. Colocalisation of FUS with SG marker poly-A binding protein (PABP) demonstrated that FUS is recruited into SGs upon osmotic stress (Figure S3A). However, we also identified both PABP- and FUS-only cytoplasmic foci (Figure S3A). hnRNPK and hnRNPA1 also form part of SGs in response to osmotic stress [16,23]. The distinct cytoplasmic granular formation and colocalisation of hnRNPA1 and hnRNPK strongly support their incorporation into SGs in human iPSC-derived motor neurons (Figure S3B). The findings of FUS-, hnRNPK- and hnRNPA1-only cytoplasmic foci, however, suggest that cytoplasmic localisation and possibly accumulation of mislocalised RBPs can occur independently of SGs, in line with recent studies [24,25].

### 2.3. RBP Nucleocytoplasmic Redistribution is Stress Specific

**Figure 3:**
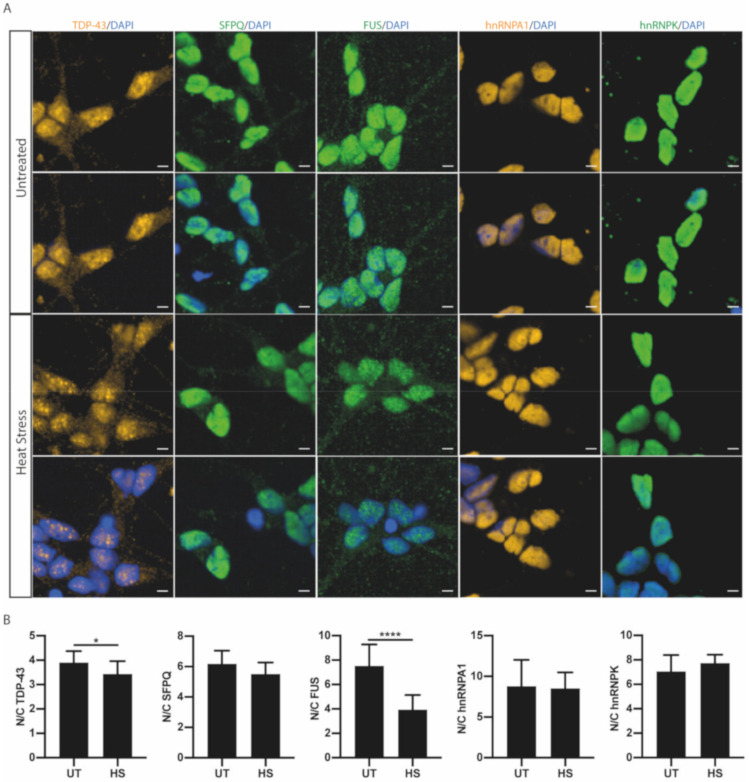
Heat stress causes stress-specific changes in nucleocytoplasmic distribution. (**A**) Representative images of TDP-43, SFPQ, FUS, hnRNPA1 and hnRNPK in iPSC-derived motor neurons in untreated and heat stress conditions, defined as 1 h at 42 °C. Scale bars = 5 μm. (**B**) Individual cell quantification of the nuclear/cytoplasmic ratio identified a significant loss of the nuclear/cytoplasmic ratio in TDP-43 and FUS under heat stress, with a small decrease in the nuclear/cytoplasmic ratio for SFPQ, but no effect on hnRNPK or hnRNPA1. Data shown are mean nuclear/cytoplasmic ratios (±SD) per well from >7000 cells from 3 control lines in 2 independent experimental repeats. *p*-value from unpaired *t*-test; * = *p* < 0.05; **** = *p* < 0.0001.

**Figure 4:**
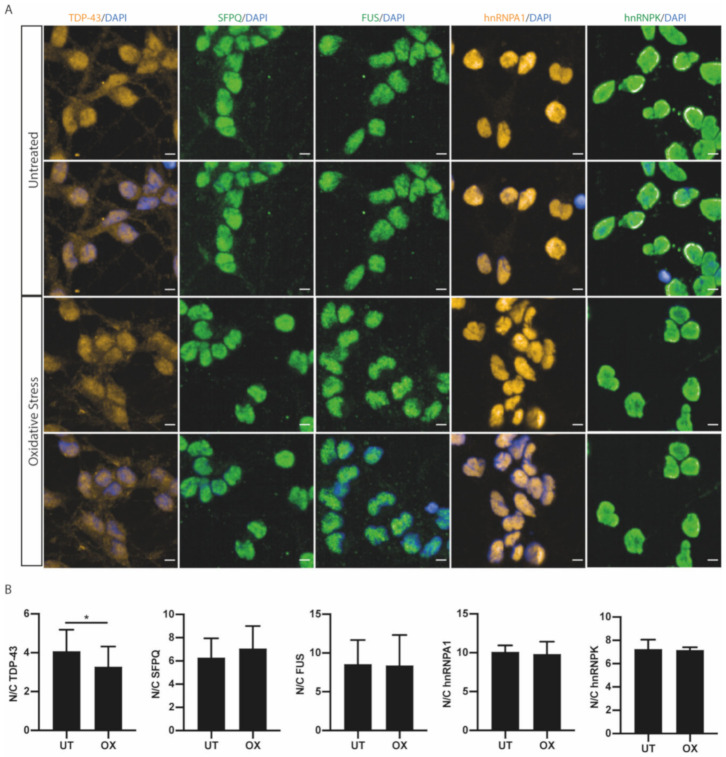
Oxidative stress causes stress-specific changes in TDP-43 nucleocytoplasmic distribution. (**A**) Representative images of TDP-43, SFPQ, FUS, hnRNPA1 and hnRNPK in iPSC-derived motor neurons in untreated and treated with 0.05 mM sodium arsenite for 1 h to stimulate oxidative stress. Scale bars = 5 μm. (**B**) Individual cell quantification of the nuclear/cytoplasmic ratio identified a significant loss of the nuclear/cytoplasmic ratio in TDP-43 with oxidative stress, with a small increase in the nuclear/cytoplasmic ratio for SFPQ, but no effect on FUS, hnRNPK or hnRNPA1. Data shown are mean nuclear/cytoplasmic ratios (±SD) per well from 3 control lines from >8000 cells for TDP-43, SFPQ and FUS and >3500 cells for hnRNPA1 and hnRNPK. *p*-value from unpaired *t*-test; * = *p* < 0.05.

We next hypothesised that the localisation responses of RBPs will also be stress-specific. We therefore analysed endogenous RBP localisation in response to heat stress [26,27,28]. MNs were subjected to 42 °C for 1 h (Figure 3A). For TDP-43, heat stress resulted in a decrease in the nuclear-to-cytoplasmic ratio, as seen with osmotic stress (Figure 3B). However, heat stress resulted in a fold change of 0.86 from untreated values, notably a significantly reduced response when compared to osmotic stress (0.33; *t*-test, *p* < 0.0001). Heat stress similarly resulted in a significant decrease in the nuclear-to-cytoplasmic ratio of FUS (Figure 3B), but with a greater effect size compared to TDP-43 (fold change from untreated: FUS = 0.51, TDP-43 = 0.86; *t*-test, *p* = 0.0002). In comparison to osmolar stress, heat stress resulted in a smaller decrease in the nuclear-to-cytoplasmic FUS ratio (fold change from untreated: osmotic stress (OSM) = 0.25, heat stress (HS) = 0.51; *t*-test, *p* < 0.0001). However, heat stress had no effect on the nuclear-to-cytoplasmic ratio of SFPQ, hnRNPA1 or hnRNPK (Figure 3B). Notably, upon heat stress, we also observed the increased formation of TDP-43 nuclear foci (Figure S4A), consistent with previous studies [29,30]. An increase in FUS nuclear foci was also observed upon heat stress; however, these granular structures were not as clearly defined as the TDP-43 foci and were more abundant (Figure S4B). Indeed, the changes in RBP localisation in response to heat stress were different from those seen in response to osmotic stress. Furthermore, those RBPs that were redistributed all showed a smaller magnitude of response, while still displaying RBP-specificity. We next examined oxidative stress (OX) using arsenite treatment. We treated MNs with sodium arsenite at 0.5 mM for 1 h to study the response of ALS-linked RBPs to oxidative stress (Figure 4A). There was no change in total protein levels following arsenite treatment (Figure S2). TDP-43 was the only ALS-linked RBP to show a significant decrease in the nuclear-to-cytoplasmic ratio (Figure 4A). The magnitude of TDP-43′s localisation response appeared stress-specific, as evidenced by a significantly different fold change compared to the other stressors (OX = 0.74, HS = 0.86, OSM = 0.33; one-way ANOVA, *p* < 0.0001). For the other RBPs examined, we observed no change in the nuclear-to-cytoplasmic ratio following oxidative stress (Figure 4A,B). Importantly, differences in effect size of RBP redistribution between different stressors may also reflect specific concentrations of the stressors used (or the specific temperature in the case of heat stress).

To explore whether there was a correlation between extrinsic stress, RBP localisation and increased cell viability, we examined cell death under the different stress conditions and recovery using a simple assay of nuclear pyknosis (data not shown). We found heat stress was the only stressor that caused cell death (22% compared to 16% in untreated (UT) conditions; *t*-test, *p* = 0.0224), with no additional cell death following 2 h of recovery.

### 2.4. TDP-43 and FUS Exhibit Slower Nuclear Relocalisation Dynamics Following Stress 

**Figure 5:**
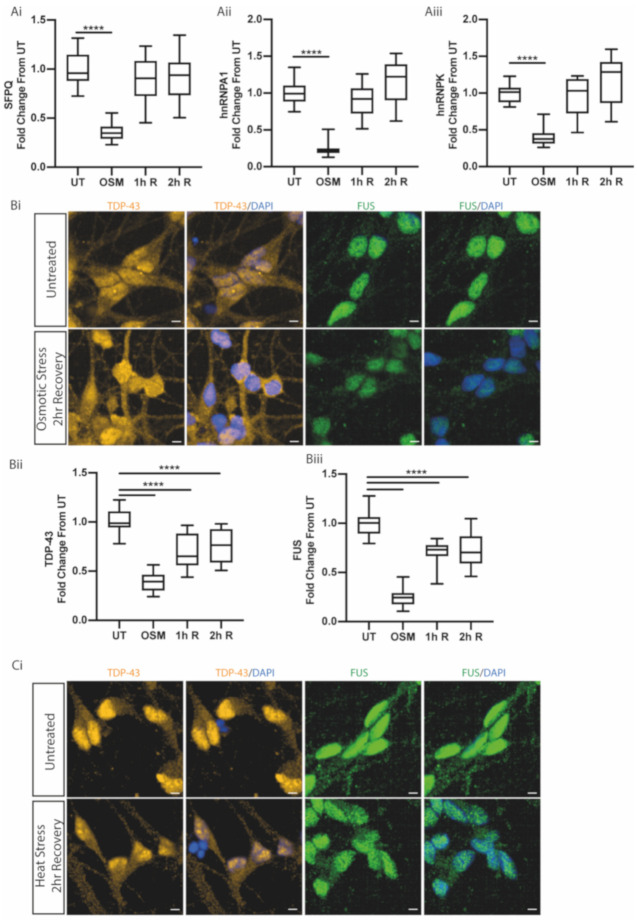

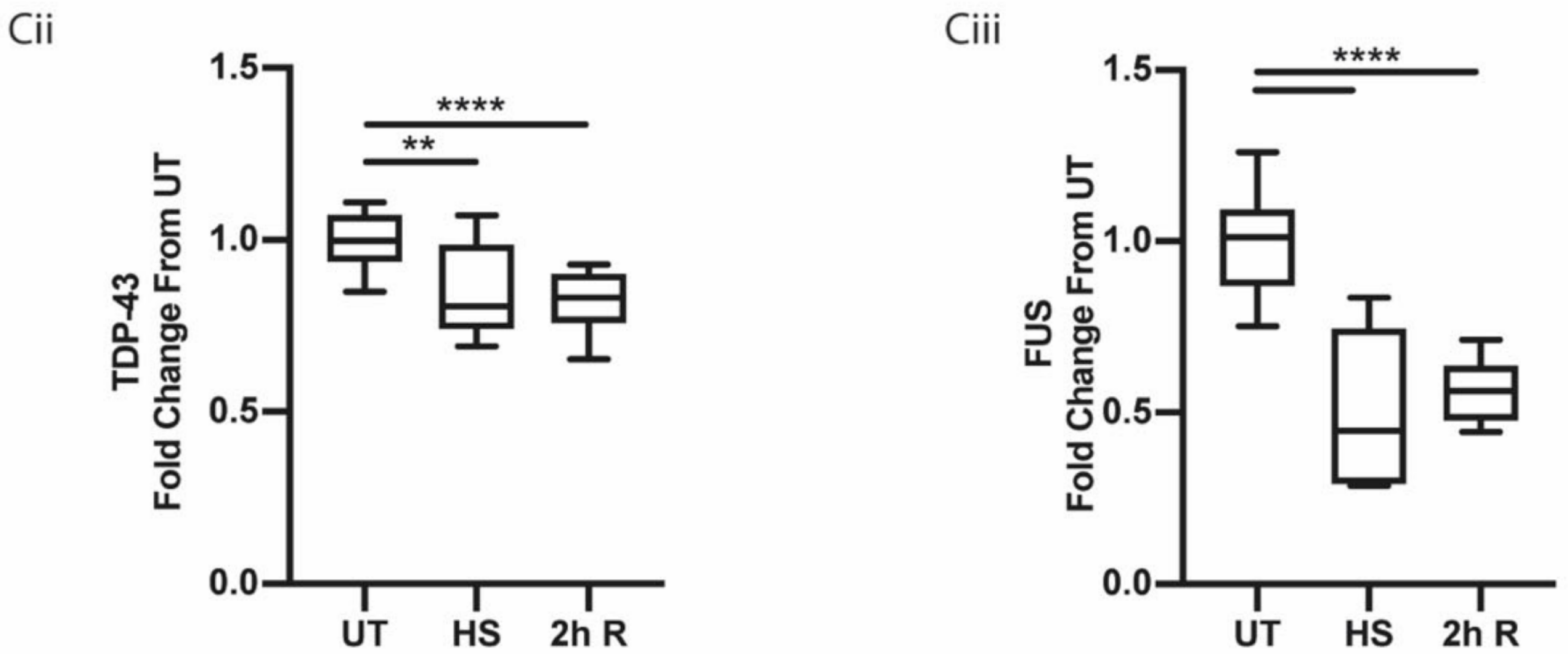
ALS-linked RBPs show different recovery rates when restoring nucleocytoplasmic distribution following stress. (**A**) Motor neurons were treated with 0.4 M sorbitol (OSM) and then allowed to recover for 1-h (1h R) and 2-h (2h R) time periods. Quantification of the RBP nuclear/cytoplasmic ratio was measured by individual cell analysis of >10,000 cells for the osmotic stress and recovery times and calculated as fold change over untreated (UT) values for each experimental repeat. Data are shown as fold change from untreated values in a box and whisker plot, plotted per well from 3 control lines in 3 independent experiments. *p*-values from an unpaired *t*-test; **** = *p* < 0.0001. (**Ai**) SFPQ showed recovery to untreated nuclear/cytoplasmic ratio by 1 h of recovery. (**Aii**) hnRNPA1 showed full recovery to untreated values by 1 h of recovery. (**Aiii**) hnRNPK showed a similar recovery profile to SFPQ and hnRNA1, returning to untreated nucleocytoplasmic distribution by 1 h of recovery. (**B**) Motor neurons were treated with 0.4 M sorbitol (OSM) and then allowed to recover for 1-h (1h R) and 2-h (2h R) time periods. Quantification is the same as above for TDP-43 and FUS. (**Bi**) Representative images of TDP-43 and FUS in motor neurons treated with 0.4 M sorbitol (OSM) and allowed to recover for 2 h. (**Bii**) TDP-43 showed continual recovery towards untreated nucleocytoplasmic distribution following 1 h and 2 h of recovery; however, it did not recover fully. (**Biii**) FUS showed a similar recovery profile to TDP-43 and showed recovery but had not recovered to untreated values following 2 h of recovery. (**C**) The nuclear/cytoplasmic ratio was calculated from motor neurons that were subjected to 42 °C for 1 h to cause heat stress (HS) and motor neurons that had recovered for 2 h following heat stress at 37 °C (2h R). Data are shown as fold change over untreated values within experimental repeats. Data are shown as a box and whisker plot, plotted per well from 3 control lines from >7000 cells for each condition in 2 independent experimental repeats. (**Ci**) Representative images of TDP-43 and FUS after 2 h of recovery following heat stress. (**Cii**) TDP-43 shows a decrease in the nuclear/cytoplasmic ratio upon heat shock, which shows no recovery after 2 h at 37 °C. (**Ciii**) FUS shows a significant fold change decrease upon heat shock, which shows no recovery after 2 h; ** = *p* < 0.01;.

We next investigated how the localisation of ALS-linked RBPs changes during recovery from stress. Following our osmotic stress paradigm, after 1 h of recovery, SFPQ, hnRNPA1 and hnRNPK had returned to basal (untreated) values (0.89, 0.91 and 0.95 respectively; no significant difference from untreated values: respective *p* values = 0.147, 0.192, 0.529) (Figure 5A). hnRNPA1 and hnRNPK recovery increased above basal values following 2 h of recovery, suggesting overcompensation (Figure 5A). In contrast, TDP-43 and FUS showed slower relocalisation kinetics, as they had not returned to basal distribution even by 2 h post-stress (Figure 5B). Immunocytochemistry 6 h post-stress showed FUS had still not returned to basal distribution (Figure S5). Similarly, following 2 h of recovery from heat stress, there was no reconstitution of nuclear TDP-43 and FUS to basal levels (Figure 5C). However, it is noteworthy that, unlike osmotic stress, the nuclear-to-cytoplasmic ratio of FUS and TDP-43 following heat stress showed no significant recovery.

### 2.5. ALS-Causing VCP Mutations Do Not Perturb Nuclear Relocalisation Dynamics of TDP-43 and FUS Following Stress

**Figure 6:**
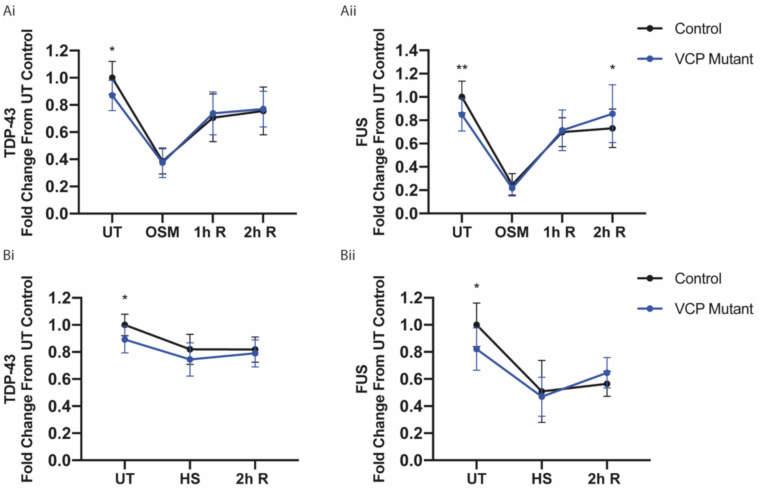
Human iPSC-derived VCP mutant motor neurons do not show perturbed TDP-43 and FUS redistribution upon stress and recovery. (**A**) Control and VCP mutant motor neurons were treated with 0.4 M sorbitol and then allowed to recover for 1-h (1h R) and 2-h (2h R) time periods. Individual cell analysis was used to quantify RBP nuclear/cytoplasmic ratio, which was then calculated as fold change over control untreated (UT) values for each experimental repeat. Data are plotted in a line graph as mean fold change (± SD) per well from >10,000 cells from 3 control lines and >20,000 cells from 4 VCP mutant lines for each experimental condition across 3 independent experiments. *p*-values displayed are from a Sidak’s multiple comparison test (* *p* < 0.05, ** *p* < 0.01). (**Ai**) For TDP-43, VCP mutant motor neurons show a loss in the nuclear-to-cytoplasmic ratio under untreated conditions but no difference in nucleocytoplasmic distribution upon osmotic stress or after recovery. (**Aii**) For FUS, VCP mutant motor neurons show a loss in the nuclear-to-cytoplasmic ratio under untreated conditions but no difference in protein localisation upon osmotic stress or after 1 h of recovery. Following 2 h of recovery, VCP mutant motor neurons show an increased degree of nuclear relocalisation. (**B**) Control and VCP mutant motor neurons were subjected to 42 °C for 1 h to cause heat stress (HS), and motor neurons recovered for 2 h following heat stress at 37 °C (2h R). Quantification was performed in the same manner as above. Data are plotted in a line graph as mean fold change (±SD) from >7000 cells from 3 control lines and >10,000 cells from 4 VCP mutant lines across 2 independent experimental repeats. *p*-values displayed are from a Sidak’s multiple comparison test (* *p* < 0.05). (**Bi**) VCP mutant motor neurons showed a loss in the nuclear-to-cytoplasmic ratio of TDP-43 under basal conditions but no difference upon heat stress or 2 h recovery. (**Bii**) VCP mutant motor neurons showed a loss in the nuclear-to-cytoplasmic ratio of FUS under basal conditions but no difference upon heat stress or 2 h recovery.

Next, we studied how the relocalisation dynamics of TDP-43 and FUS were affected by ALS-causing VCP mutations. Previously, we had characterised pathogenic phenotypes in a human iPSC model of VCP-mutation related ALS (VCP^R155C^ and VCP^R191Q^) including TDP-43 and FUS nuclear-to-cytoplasmic mislocalisation [6,22]. We used this well-characterised model to investigate how VCP mutations affect the subcellular localisation of TDP-43 and FUS upon the aforementioned cellular stress and recovery paradigms in human MNs. In untreated conditions, VCP mutant MNs displayed a loss of the nuclear-to-cytoplasmic ratio of both TDP-43 and FUS (Figure 6A,B). Despite this difference in the untreated condition, TDP-43 and FUS showed no difference in the degree to which nuclear-to-cytoplasmic ratio was reduced upon osmotic stress (Figure 6A). When examining recovery from osmotic stress, VCP mutant MNs restored nuclear TDP-43 localisation at the same rate as control MNs (Figure 6(Ai)). Similarly, restoration of FUS distribution occurred at the same rate following 1 h of recovery; however, VCP mutant MNs seemed to overcompensate following 2 h of recovery (Figure 6(Aii)). When examining heat stress, control and VCP mutant MNs displayed no difference in the nucleocytoplasmic redistribution of TDP-43 and FUS. Following 2 h recovery, both control and VCP mutant MNs failed to recover nuclear TDP-43 or FUS to their respective basal levels (Figure 6B).

## 3. Discussion

RBPs have a central role in neurodegeneration, with their mislocalisation representing a hallmark of ALS and frontotemporal lobar degeneration (FTLD), a related disorder. Here, we deliberately chose RBPs implicated in neurodegeneration, including TDP-43, SFPQ, FUS, hnRNPA1 and hnRNPK [4,5,6,31,32]. Studying the cellular response to stress is crucial for understanding potential mechanisms of RBP localisation and aggregation. Previously in the literature, studies have shown that cellular stress has the potential to alter the subcellular localisation of these RBPs; however, this has not yet been systematically tested in a human motor neuronal model [11,12,13,14,15,16]. Furthermore, previous studies did not addressed the dynamics of RBP relocalisation to the nucleus following stressor removal and, therefore, whether this might contribute to exacerbating RBP mislocalisation pathology. To this end, our study has systematically investigated how diverse cellular stressors affect key neurodegeneration-related RBPs in a human neuronal model.

Osmotic stress resulted in a decrease in the nuclear-to-cytoplasmic ratio of all five RBPs studied in our human MN model (Figure 2A,B). As whole-cell analysis of protein levels upon osmotic stress showed no change, we can conclude that the decrease in the nucleocytoplasmic ratio can be accounted for by subcellular redistribution and is not attributable to protein degradation. When examining the immunocytochemistry images for TDP-43, SFPQ and FUS, there appears to be a loss in intensity upon osmotic stress, which may explained by a greater redistribution of the protein into the neuronal process, thereby resulting in a lower detectable intensity in the nucleus and soma. Previously, osmotic stress has been shown to relocalise TDP-43, FUS, hnRNPA1 and hnRNPK into the cytoplasm, where inclusions identified as stress granules are formed [11,12,13,16,23,33]. The mechanism by which cytoplasmic aggregates form is unknown; however, an attractive hypothesis is that an alteration in the dynamics of liquid–liquid phase separation (LLPS) of RBPs contributes to the formation of these pathological inclusions [34,35]. Recently, RBP translocation and stress granule incorporation have been shown to be uncoupled [17]. Despite our findings of a stress-related decrease in nuclear-to-cytoplasmic ratio of RBPs, the effect size differed between RBPs, suggesting individualized RBP-specific responses. Recent studies have shown that TDP-43 and FUS undergo nuclear export by passive diffusion, whereas subcellular distribution changes of hnRNPA1 are achieved by hyperphosphorylation to reduce the rate of nuclear import [17,33,36]. Further studies are required to understand how each RBP undergoes nuclear export upon osmotic stress. It is also noteworthy that RBPs can possibly undergo nuclear degradation [37].

Differences between ALS-linked RBPs in their response to osmolar stress were further highlighted when studying recovery from this insult, and thus mechanisms of nuclear import. SFPQ, hnRNPA1 and hnRNPK showed similarities in their recovery profiles following extrinsic stress. However, even these three RBPs possess multiple unique nuclear localisation signals (NLS) and distinct nuclear import pathways. SFPQ has shown to have two weak nuclear localisation signals (NLSs), one resembling a classical NLS and the other a bipartite basic NLS, that are both required for complete transport into the nucleus [38]. hnRNPK also has two NLSs, a classical, bipartite basic NLS and a domain called the KNS, a novel nuclear shuttling domain that uses a separate import pathway [38,39]. hnRNPA1has a different non-canonical NLS, a PY-NLS, and likely operates through a distinct nuclear import pathway [40]. Temporal similarities in recovery amongst the three RBPs suggest each of these nuclear import pathways can operate at similar rates. Interestingly, FUS and TDP-43 fail to recover their basal localisation even 2 h following stress. This similarity cannot be explained by use of the same nuclear import pathway as TDP-43 has a canonical NLS utilising karyopherin-α and karyopherin-β1, whilst FUS contains a PY-NLS which undergoes nuclear import via karyopherin-β2 [41,42]. Despite clear differences, it is noteworthy that all RBPs follow an initial fast phase of recovery. This suggests that there is an initial, potentially more generalized, cytoplasmic to nuclear transport mechanism, which changes once a threshold is reached. Interestingly it was TDP-43 and FUS that showed slow relocalisation kinetics, as two of the major pathological hallmarks of ALS [4,6]. Although we observed a correlation between TDP-43 and FUS mislocalisation and cell death, this likely reflects the specific response to the stressor being examined; further studies should investigate whether viability is in any way dependent on TDP-43 and FUS localisation or not.

TDP-43 and FUS are further implicated when examining stress-specific RBP responses in the MNs. Unlike the widespread effect of osmotic stress on RBP nucleocytoplasmic ratios, heat stress resulted in a more selective decrease of the nuclear-to-cytoplasmic ratio of TDP-43 and FUS, and oxidative stress only altered TDP-43 distribution. The increased propensity of TDP-43 and FUS to be present in the cytoplasm under multiple stressors may encourage an aggregation-prone environment. In human MNs, it has been shown that an increase of cytoplasmic TDP-43 is sufficient to drive LLPS of TDP-43 independently of stress granule formation [25]. It is therefore notable that when examining recovery from heat stress, TDP-43 and FUS show no recovery in nucleocytoplasmic ratio 2 h after stressor removal (Figure 5C). Therefore, there is not only a reduction in the nuclear-to-cytoplasmic ratio of FUS and/or TDP-43 across different stressors but also an increased duration of their mislocalisation due to comparatively slower nuclear relocalisation dynamics. Cellular ageing will further exacerbate this phenomenon, as it results in the reduced efficiency of nuclear import [43,44,45]. Studies examining the stress response in the context of ALS-causing gene mutations in TDP-43 and FUS have mostly focused on the incorporation of the RBPs into stress granules, as aberrant stress granules have been hypothesised to contribute to pathological aggregation in ALS. Mutations in TDP-43 have been shown to influence stress granule dynamics including assembly, disassembly, size and the amount of TDP-43 within the stress granule [46]. Mutations in FUS have been shown to cause FUS cytoplasmic mislocalisation, which is linked to increased recruitment into stress granules following heat and oxidative stress [47]. Furthermore, ALS mutations have also been shown to cause nucleocytoplasmic transport defects, directly impacting the nuclear import mechanism responsible for restoring spatial homeostasis of RBPs [48]. This includes mutations in the nuclear localisation signal of both FUS and hnRNPA1 and the ALS-causing hexanucleotide repeat in the gene C9ORF72 [48]. The ALS-causing VCP mutations R155C and R191Q in this study did not show nucleocytoplasmic transport defects following cellular stress, despite a reduction in their nuclear-to-cytoplasmic ratios under basal conditions. ALS results from a wide variety of genetic insults, and multiple mechanisms are involved in the disease pathogenesis, accounting for the differences in nucleocytoplasmic defects [49]. Taken together, our findings suggest that the basal TDP-43 and FUS mislocalisation observed in VCP mutant MNs is not (at least initially) caused by failure of their nuclear relocalisation following cellular stress. Our findings specifically demonstrate that TDP-43 nuclear loss is a widespread consequence across multiple stress-inducing pathways. Disruption in TDP-43 localisation will affect the TDP-43 autoregulation pathway, with a reduced nuclear TDP-43 function producing a positive feedback loop producing increased levels of cytoplasmic TDP-43 [48,50,51]. Indeed, ALS-causing mutations likely lead to cellular stress through multiple possible pathways, inducing changes in the nucleocytoplasmic distribution of RBPs [22,52,53].

In summary, our study demonstrates that cellular stress results in the mislocalisation of different RBPs to different extents in human MNs. Against this background, the nucleocytoplasmic redistribution of RBPs appears to be both RBP- and stress-specific. Importantly, we reveal that TDP-43 and FUS, two of the most recognised RBPs in ALS pathogenesis, exhibit delayed nuclear relocalisation as compared to SFPQ, hnRNPA1 and hnRNPK. An increased duration of TDP-43 and FUS within the cytoplasm after stress may render the environment more aggregation-prone, which may be poorly tolerated in the context of ALS and related neurodegenerative disorders. Such molecular heterogeneity when comparing across the five aforementioned RBPs argues for tight regulation of their spatial distribution. It is crucial to understand how disruption of these homeostatic processes specifically relates to RNA metabolism and protein homeostasis in the context of neurodegeneration. Further understanding of the molecular phases of pathogenesis in a cell-type-specific fashion will help to identify tractable therapeutic targets for devastating diseases such as ALS and related disorders.

## 4. Materials and Methods

Detailed methods are provided in the Supplementary Materials.

### 4.1. Ethics Statement

Informed consent was obtained from all patients and healthy controls in this study. Experimental protocols were all carried out according to approved regulations and guidelines by UCLH’s National Hospital for Neurology and Neurosurgery and UCL’s Institute of Neurology joint research ethics committee (09/0272).

### 4.2. Stress Treatments

To induce osmotic stress, sorbitol was added to the medium to add an additional osmolarity of 0.4 M. To allow for recovery, the sorbitol medium was replaced with fresh untreated medium for the set period of time. To induce oxidative stress, 0.5 mM of sodium arsenite (Sigma, Saint Louis, MO, USA) was added to the medium. To induce heat stress, cells were placed in an incubator at 42 °C and 5% carbon dioxide for 1 h. For heat stress recovery, cells were returned to an incubator at 37 °C and 5% carbon dioxide for 2 h.

### 4.3. Immunocytochemistry

For immunocytochemistry, cells were fixed in 4% paraformaldehyde in PBS for 15 min at room temperature (RT). Cells were permeabilised and nonspecific antibody binding was blocked using 0.3% Triton-X containing 5% bovine serum albumin (BSA) (Sigma) in PBS for 60 min. Primary antibodies used were SMI-32 (BioLegend, San Diego, CA, USA; 801,701; mouse; 1:1000), ChAT (Millipore, Burlington, MA, USA; AB144P; goat; 1:100), β-III-tubulin (abcam, Cambridge, UK; ab41489; chicken; 1:1000), TDP-43 (ProteinTech, Rosemont, IL, USA; 12892-1-AP; rabbit; 1:400), SFPQ (abcam, Cambridge, UK; ab11825; mouse; 1:400), FUS (Santa Cruz, Santa Cruz, CA, USA; sc-47711; mouse; 1:200), hnRNPA1 (Cell Signaling, Danvers, MA, USA; 8443S; rabbit; 1:500) and hnRNPK (Santa Cruz, Santa Cruz, CA, USA; sc-28380; mouse; 1:500). Primary antibodies were made up in 5% BSA and then applied overnight at 4 °C. Cells were washed with PBS 2 times, and secondary antibody was added using a species-specific Alexa Fluor-conjugated secondary antibody (Life Technologies, Carlsbad, CA, US) at 1:1000 dilution in 5% BSA for 90 min at RT in the dark. Cells were washed once in PBS containing 4′,6-diamidino-2-phenylindole (DAPI) nuclear stain (1:1000) for 10 min. Following a second wash in PBS, cells were left in PBS in the dark.

### 4.4. Image Acquisition and Analysis

Images were acquired using the Opera Phenix High-Content Screening System (PerkinElmer, Waltham, MA, USA). Images were acquired with a 40× or 63× objective as confocal z-stacks with a z-step of 1 μm and processed to obtain a maximum intensity projection. A minimum of 12 fields of view were taken for each well. Images were analysed using the Columbus Image Analysis System (PerkinElmer). The nuclear/cytoplasmic ratio of proteins was analysed on a cell-by-cell basis. A DAPI mask was applied to define the nucleus, and a trained machine learning feature selected neurons, removing dead cells and double cells based on nuclei properties. For each individual cell, an average nuclear intensity of the protein was measured. For the cytoplasmic measurement, a 1.5 µm cytoplasmic region was defined around the nucleus within a mask defined by a cytoplasmic protein, and an average intensity was measured. A ratio of the nuclear to cytoplasmic intensity measurements was calculated for each individual cell and then averaged across the well to give the main experimental outcome.

## Supplementary Materials

The following are available online at https://www.mdpi.com/1422-0067/21/21/8346/s1.
