# Supplementary material for: Stress-Specific Spatiotemporal Responses of RNA-Binding Proteins in Human Stem Cell-Derived Motor Neurons
Source: Int J Mol Sci. 2020 Nov 6;21(21):8346. doi: 10.3390/ijms21218346 (PMC7664327; doi:10.3390/ijms21218346)
Supplement: Supplementary file 1 [file ijms-21-08346-s001.pdf]

SUPPLEMENTARY MATERIAL

Stress-Specific Spatiotemporal Responses of RNA Binding Proteins in Human Stem Cell-derived Motor Neurons

Authors: Jasmine Harley[1,2], Rickie Patani[1,2]

SUPPLEMENTARY FIGURES

A

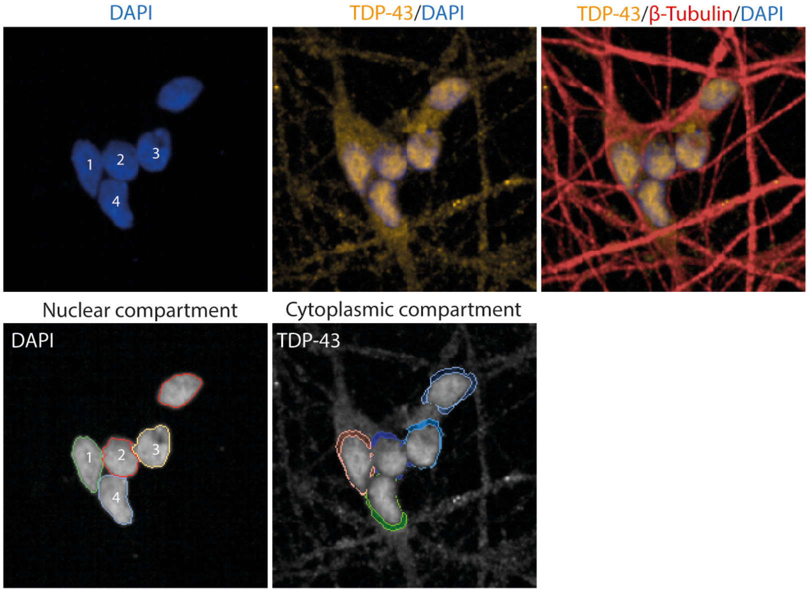

B

Cell	1	2	3	4
**Mean Nuclear TDP-43**	671.522	568.694	627.534	654.085
**Mean Cytoplasmic TDP-43**	274.889	217.146	291.908	296.967
**N/C Ratio**	2.44289000	2.61894762	2.14976636	2.20255113

Supplementary Figure 1. The analysis method used to quantify the nuclear to cytoplasmic ratio. A) Example images of the subcellular compartmentation of neurons using TDP-43 immunolabeling as an example with DAPI and neuronal marker β-III-tubulin. The nuclear compartment is defined by the DAPI nuclear stain. The cytoplasmic compartment is defined within a cytoplasmic mask restricted to 1.5µm around the nucleus. B) An example quantification of the nuclear to cytoplasmic ratio using the mean intensity of each nuclei divided by the mean intensity of its corresponding cytoplasmic compartment.

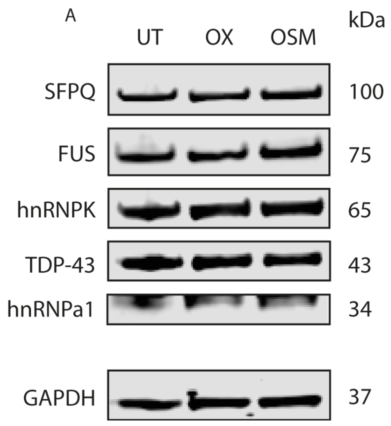

Supplementary Figure 2. Western blot analysis of whole cell protein levels upon osmotic and oxidative stress. A) Immunoblot of TDP-43, SPFQ, FUS, hnRNPA1 and hnRNPK showed that overall levels are unchanged upon osmotic and oxidative stress compared to GAPDH in control motor neurons.

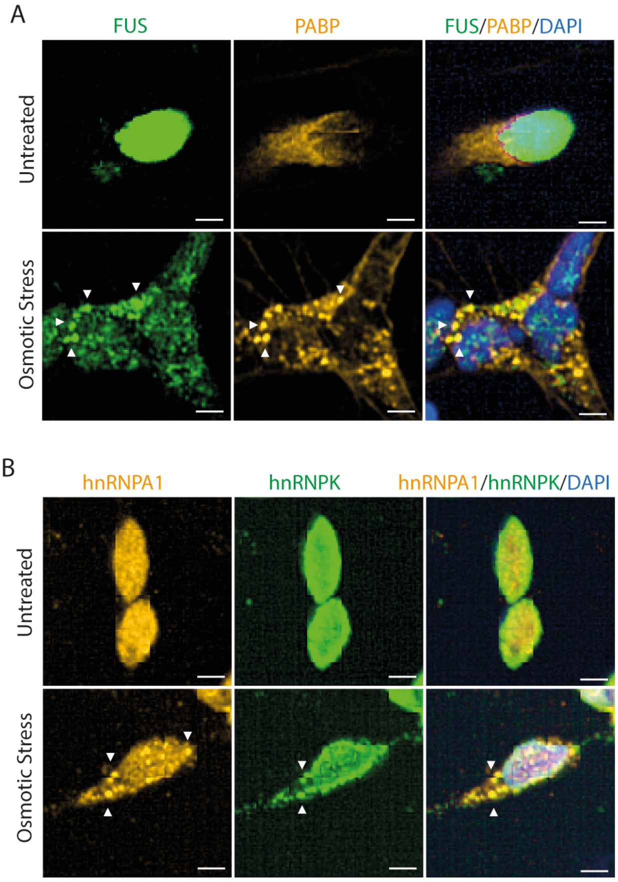

Supplementary Figure 3. Cytoplasmic foci formation upon osmotic stress. A) FUS cytoplasmic foci upon osmotic stress were identified by immunocytochemistry. Stress granule formation was identified by the formation of PABP positive foci. FUS cytoplasmic foci showed both PABP positive and PABP negative formation. Scale bars = 5μm. B) Upon osmotic stress hnRNPA1 and hnRNPK formed cytoplasmic foci, that displayed both individual and colocalised immunolabeling, with similar immunolabeling patterns to that seen of PABP and FUS. Scale bars = 5μm.

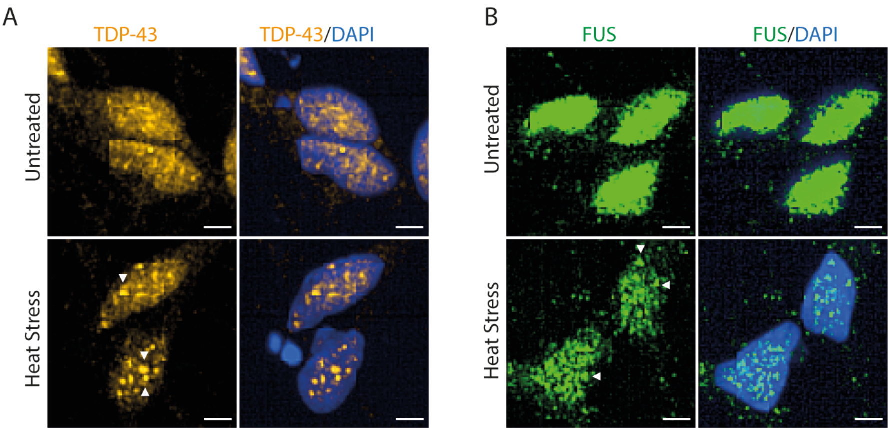

Supplementary Figure 4. Nuclear foci formation upon heat stress. A) Immunolabeling of TDP-43 nuclear foci in motor neurons under heat stress. Scale bars = 5μm. B) Images show motor neurons with FUS nuclear punctate under heat stress conditions. Scale bars = 5μm.

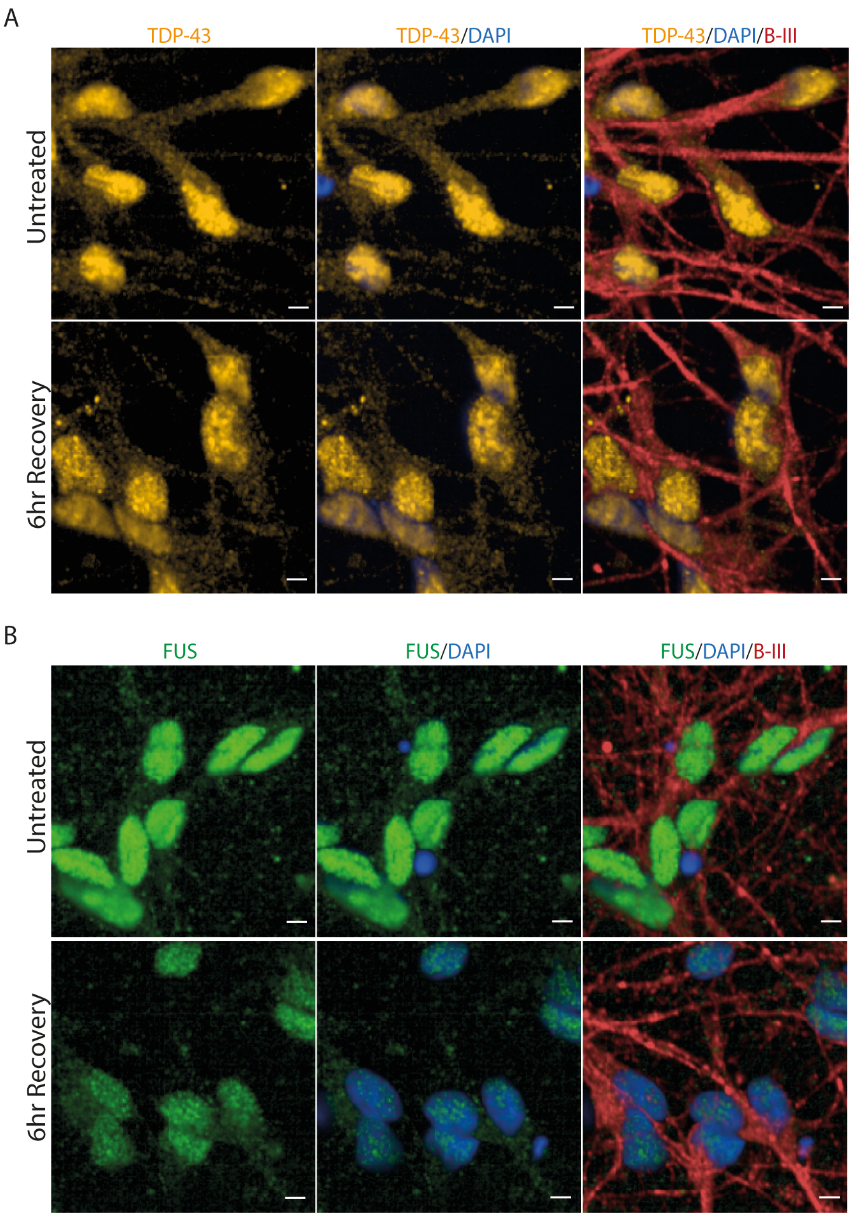

Supplementary Figure 5. TDP-43 and FUS immunolabeling 6 hours recovery following osmotic stress. A) TDP-43 shows cytoplasmic recovery following 6 hours of recovery following osmotic stress. Scale bars = 5μm. B) FUS does not show complete nuclear relocalisation following 6 hours of recovery following osmotic stress. Scale bars = 5μm.

SUPPLEMENTARY METHODS

Human Fibroblasts and iPSC

Dermal fibroblasts were cultured in OptiMEM +10% FCS medium. For iPSC generation the following episomal plasmids were transfected: pCXLE hOct4 shp53, pCXLE hSK, and pCXLE hUL (Addgene) (Okita et al., 2011). Two of the control lines used (control 2 and control 3) are commercially obtainable and were purchased from Coriell (cat. Number ND41866*C) and ThermoFisher Scientific (cat. number A18945). Four VCP mutant lines were used, 2 clones from a patient with a VCP R155C mutation, and 2 clones from a patient with a VCP R191Q mutation.

Cell culture and motor neuron differentiation

Induced PSCs were maintained on Geltrex (Life Technologies) with Essential 8 Medium media (Life Technologies), and passaged using EDTA (Life Technologies, 0.5mM). All cell cultures were maintained at 37°C and 5% carbon dioxide. iPSCs were first differentiated to neuroepithelium by plating to 100% confluency in medium consisting of DMEM/F12 Glutamax, Neurobasal, L-Glutamine, N2 supplement, nonessential amino acids, B27 supplement, β-mercaptoethanol (all from Life Technologies) and insulin (Sigma). The cells then underwent a sequential treatment with small molecules from day 0-7 was as follows: 1μM Dorsomorphin (Millipore), 2μM SB431542 (Tocris Bioscience), and 3.3μM CHIR99021 (Miltenyi Biotec). At day 7, the precursors were patterned for 7 days with 0.5μM retinoic acid (Sigma) and 1μM Purmorphamine (Sigma) to respectively caudalise and ventralise precursors to the pMN domain. At day 14, spinal cord MN precursors were treated with 0.1μM Purmorphamine for a further 4 days before being terminally differentiated in 0.1μM Compound E (Enzo Life Sciences) to promote cell cycle exit. Throughout the neural conversion and patterning phase (D0-18) the neuroepithelial layer was enzymatically dissociated twice (at D4-5 and D10-12) using dispase (GIBCO, 1 mg per ml). For final plating the neural precursors were dissociated with Accutase (Life Technologies) and plated onto a 96 well plate (Falcon) on polyethylenimine (PEI) (2.2mg/ml in 0.1M of sodium borate) (Sigma) and Geltrex coated plates. After 6 days of terminal differentiation, cells were treated and fixed in 4% paraformaldehyde for immunolabeling.

Western blot analysis

Protein levels of RBPs were assessed in whole cells in basal conditions and upon stress. Cells were subjected to previously described stress treatments prior to protein extraction. The cells were lysed and proteins were extracted with RIPA disruption. Total protein concentration was quantified using BCA assay (Pierce). Equal amounts of protein samples were then loaded onto a gel and separated by SDS PAGE and transferred onto a nitrocellulose membrane. Samples were then blocked with PBS, 0.1% Tween, 5% dry milk powder at RT for one hour followed by primary antibody incubation overnight at 4 °C. The following antibodies were diluted in PBS 5% BSA; TDP-43 (ProteinTech; 12892-1-AP; rabbit; 1:1000), SFPQ (Abcam; 11825; mouse; 1:250), FUS (Santa Cruz; sc-47711; mouse; 1:500) hnRNPA1 (Cell Signaling; 8443S; rabbit; 1:1000), hnRNPK (Santa Cruz; sc-28380; mouse; 1:1000), GAPDH (Life Technologies; AM43000; mouse; 1:5000). For detection, membranes were incubated with species-specific near infra-red fluorescent antibodies (IRDye, Licor) at RT for one hour and imaged using an Odyssey Fc Imaging System (Licor).

Statistical analysis

An unpaired two-tailed student's t-test was used when comparing between two individual groups. A one way anova, Brown-Forsythe anova or 2way ANOVA with Sidak's multiple comparison test was used when comparing differences between the means of two or more groups. Statistical analysis was conducted by Prism 8. A p value 0.05 or below was considered to be statistically significant (*$p<0.05$, **$p<0.01$, ***$p< 0.001$, ****$p<0.0001$).
